# Modelling of the dynamic polarizability of macromolecules for single-molecule optical biosensing

**DOI:** 10.1038/s41598-022-05586-0

**Published:** 2022-02-07

**Authors:** Larnii S. Booth, Eloise V. Browne, Nicolas P. Mauranyapin, Lars S. Madsen, Shelley Barfoot, Alan Mark, Warwick P. Bowen

**Affiliations:** 1 ARC Centre for Engineered Quantum Systems (EQUS), School of Mathematics and Physics, The University of Queensland, Brisbane, Australia; 2 School of Chemistry and Molecular Biosciences, The University of Queensland, Brisbane, Australia

**Keywords:** Biophysics, Computational biophysics, Molecular biophysics, Nanoscale biophysics, Single-molecule biophysics, Proteins, Biological physics, Optical physics, Nanophotonics and plasmonics, Computational science

## Abstract

The structural dynamics of macromolecules is important for most microbiological processes, from protein folding to the origins of neurodegenerative disorders. Noninvasive measurements of these dynamics are highly challenging. Recently, optical sensors have been shown to allow noninvasive time-resolved measurements of the dynamic polarizability of single-molecules. Here we introduce a method to efficiently predict the dynamic polarizability from the atomic configuration of a given macromolecule. This provides a means to connect the measured dynamic polarizability to the underlying structure of the molecule, and therefore to connect temporal measurements to structural dynamics. To illustrate the methodology we calculate the change in polarizability as a function of time based on conformations extracted from molecular dynamics simulations and using different conformations of motor proteins solved crystalographically. This allows us to quantify the magnitude of the changes in polarizablity due to thermal and functional motions.

## Introduction

**Figure 1:**
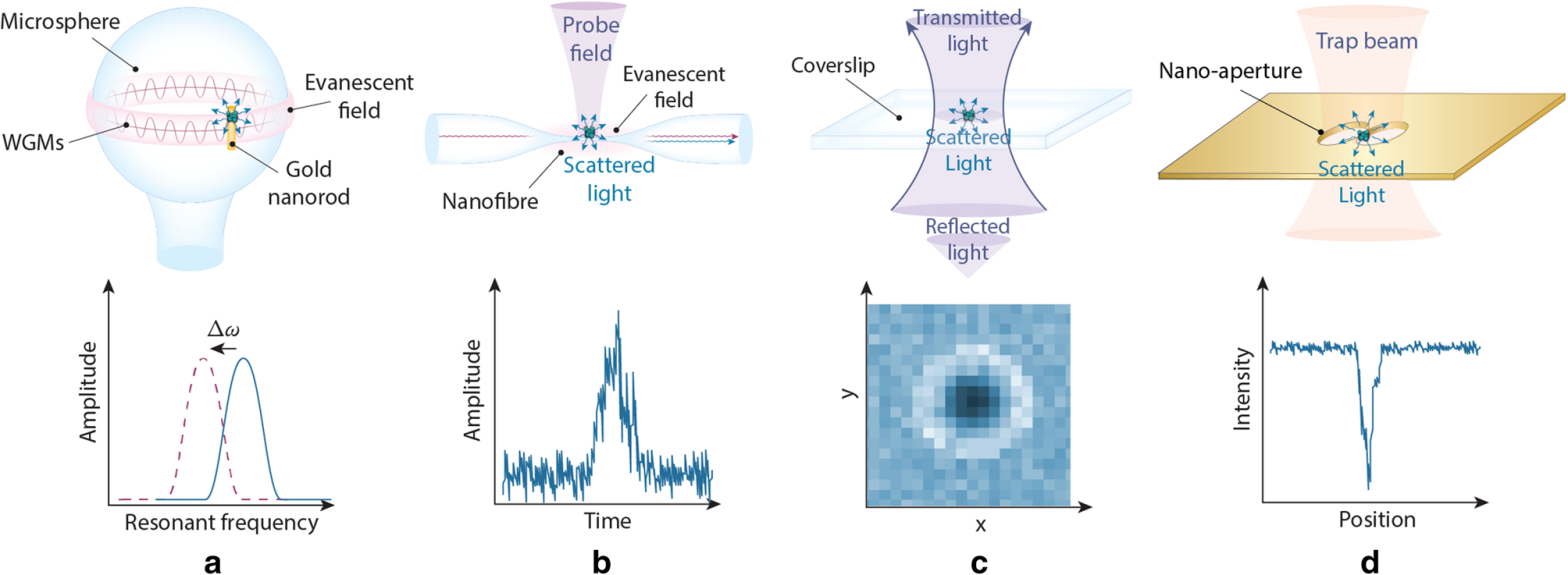
Single-molecule biosensors and their corresponding signals measured. (**a**) Optoplasmonic sensors use a shift in a cavity resonance frequency to detect the presence of a molecule^5^, (**b**) dark-field heterodyne microscopy collects the scattered field from a biomolecule and measures the beat-note of this field with light at a slightly different frequency^4^. (**c**) Interferometric scattering microscopy (iSCAT) uses the interference between the light reflected from the glass cover slip and the light scattered from the molecule to construct an image of the molecule^6^. (**d**) Plasmonic tweezers monitor the intensity of light transmitted through a sub-diffraction-limited hole in a plasmonic structure as a biomolecule enters the hole^11^.

Dielectric interactions between light and matter are widely used in biological sensing, with applications ranging from phase contrast microscopy^1^ to dynamic light scattering^2,3^. In recent years a significant focus has been to extend the applications of these interactions into the regime of single macromolecules, with particular interest in label-free identification and dynamical tracking^4–6^. This is challenging because dielectric interactions are exceedingly weak for particles that are smaller than the optical wavelength, with the cross-section of dipole scattering scaling as the particle radius to the power of six^7^. Nevertheless, several techniques have been developed that are able to resolve macromolecules with sizes down to a few nanometers, far below the scale of the optical wavelength. Figure 1 illustrates a number of these techniques, including optical cavity or plasmonic resonance enhanced sensors^8–13^, dark-field heterodyne microscopy^4,14–16^, interferometric scattering microscopy^6,17–19^, and plasmonic optical traps^20–22^.

It has been shown that single-molecule sensors which use dielectric interactions provide a signal that is highly correlated to the mass of the macromolecule being measured, independent of the molecular structure^6,23^. This enables a powerful approach for analysing molecules, molecular assembly, and chemical reactions, termed *light photometry*^24^. On the other hand, it has also been shown that the signal measured can also contain structural information about the molecule^25^, allowing label-less probing of structural dynamics. While not mutually inconsistent, these contrasting observations raise questions regarding how the conformation of a macromolecule affects the strength of the dipole interaction, and whether measurements at the single-molecule level could be used to provide a quantitative understanding associated with the structural dynamics of biological processes such as protein folding^26,27^ and enzyme catalysis.

Previous models of dielectric light-matter interactions which have been applied to single-molecule sensors have generally treated the molecule as a homogeneous material^4,23^, or as a compound structure containing several such homogeneous components^25^. These approaches are limited to prediction of large-scale effects such as motion of the molecule out of the sensing region, differential motion of model components, or assembly of multiple molecules into larger structures. They are insensitive to inhomogeneities within the molecule and to changes in its structure. Here, we introduce a method to calculate the dynamic polarizability directly from the atomistic structure of the molecule. The basis of our approach is the interactive dipole model of Applequist et al.^28^. We combine this model with efficient computational techniques to allow the polarizability of molecules containing more than one hundred thousand atoms to be calculated. We apply our method both to time-domain molecular dynamic simulations, tracking the thermally agitated fluctuations of the polarizability of Bovine Serum Albumin (BSA) as a function of time, and to predict the change in polarizability as the molecular motors ATPase and 26S proteasome change their conformations. We briefly discuss the extent to which differences in polarizability stemming from the changes in conformation could be resolved using state-of-the art single molecule light-scattering measurements. Applequist-type approaches are already used to calculate polarizable force fields in molecular dynamics simulations^29–34^. These simulations generally take account of both intra-molecule electrostatic forces and dynamic polarization forces. By contrast, our approach deals only with the dynamic polarization in response to high frequency optical excitation ($\sim 10^{12}$ Hz). The aim is to provide a simple method to determine the light scattering properties of macromolecules that is easily accessible to the single molecule sensing community. In line with this aim, we have made the code developed to calculate the dynamic polarizability openly available^35^. We believe that our approach will find applications translating the measured optical signals that arise from the dipole interaction into a quantitative understanding of the structural dynamics of macromolecule, which could be applied to better understand important biological processes such as protein folding and enzyme dynamics.

## Theory

**Figure 2:**
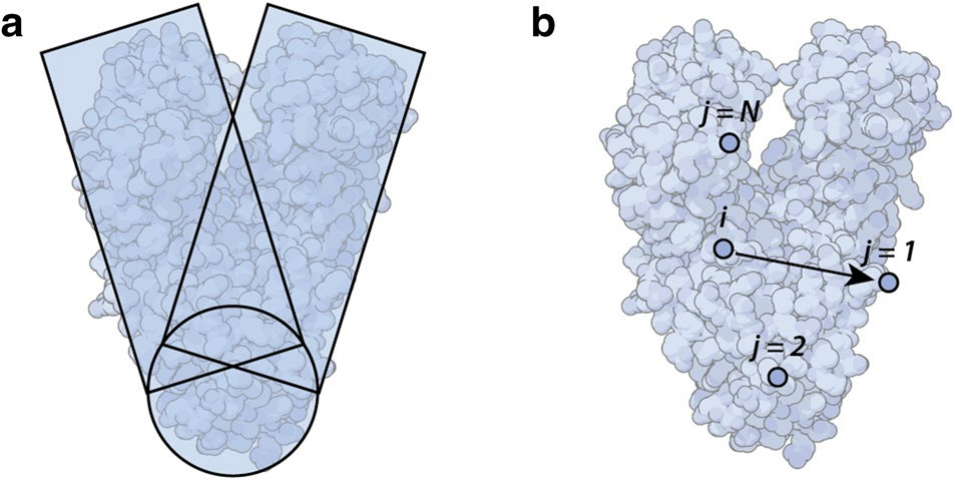
(**a**) Bulk model versus (**b**) atomistic dielectric model. [Protein Data Bank ID (PDBID): 4YFU], illustrative purposes only.

Many of the single-molecule sensors in Fig. 1 produce a signal via the dipole interaction between the molecule and light. In this case the size of the signal detected depends on the power scattered from the molecule. The mean power scattered, $\langle P_{sc} \rangle$, is:

1 $$\begin{aligned} { \langle P_{sc} \rangle } = \frac{c\varepsilon _0k^4}{96\varvec{\pi }{{n}_m}^3} ||\varvec{\mu }_{ex}||^2, \end{aligned}$$

where *c* is the speed of light; $\varepsilon _0$ is the vacuum permittivity; $n_m$ is the refractive index of the medium surrounding the molecule; and $k = 2\pi /\lambda$, where $\lambda$ is the wavelength of the incident light and $\varvec{\mu }_{ex}$ is the excess dipole moment^7^. The excess dipole moment is given by $\varvec{\mu }_{ex} = \varvec{\mu }_{mol} - \varvec{\mu }_{m}$, where $\varvec{\mu }_{mol}$ is the molecular dipole moment and $\varvec{\mu }_{m}$ is the dipole moment of the surrounding medium displaced by the molecule. It depends on the applied electric field $\varvec{E(r)}$ via the relationship

2 $$\begin{aligned} \varvec{\mu }_{ex} = \varvec{\alpha }_{ex} \cdot \varvec{E(r)}, \end{aligned}$$

where $\varvec{\alpha }_{ex}$ is the excess polarizability tensor. Therefore, the power scattered from the molecule depends on its excess polarizability. For the simple case that the molecule is an isotropic sphere, the scattered power is given by the well-known expression^4^:

3 $$\begin{aligned} \langle P_{sc} \rangle = \frac{\sigma }{8}I_{in} = \frac{k^4\bar{\alpha }_{ex}^2}{48\pi \varepsilon _m^2}I_{in}, \end{aligned}$$

where $I_{in}$ is the intensity of the input field in $W/m^2$; $\sigma$ is the scattering cross section of the molecule; $\varepsilon _m$ is the permittivity of the surrounding medium; and $\bar{\alpha }_{ex}$ is the average polarizability, defined as $\bar{\alpha }_{ex}$ = $\bar{\alpha }_{av}$ - $\bar{\alpha }_{m}$, where $\bar{\alpha }_{av}$ is the average polarizability of the molecule and $\bar{\alpha }_{m}$ is the average polarizability of the surrounding medium.

In the field of single-molecule biosensing, the biomolecule is often approximated as a simple dielectric sphere^4^ or collection of bulk shapes^25^ (see Fig. 2a). The polarizability and scattered power are then calculated from bulk properties such as the refractive index of the molecule in solution. However, bulk constants such as refractive index break down at the nanometre scale of single biomolecules. Therefore, alternative methods that account for the atomic structure of molecules are required to accurately calculate their polarizability.

This paper develops an atomistic method based on the interactive dipole model devised by Applequist et al.^28^ The model assumes that each molecule is a collection of *N* atoms, each at fixed spatial coordinates. In the ideal case, this method evaluates both the dipole induced in each atom by an external electric field $\varvec{E}$, and the dipole moment induced by the interaction of each atom with every other atom in the molecule (Fig. 2b). The induced dipole moment of the entire molecule ($\varvec{\mu }_{mol}$) is the sum of the induced dipole moments of each atom, $\varvec{\mu }_{i}$:

4 $$\begin{aligned} {\varvec{\mu }}_{{mol}} = \sum _{i=1}^N\varvec{\mu }_{i}. \end{aligned}$$

The induced dipole moment for each atom *i* is a function of the applied electric field given by

5 $$\begin{aligned} \varvec{\mu }_{i}(\varvec{E}(\varvec{r})) = {\alpha }_i \left[ \varvec{E}(\varvec{r}_{i}) - \sum _{j\ne i}^N {\mathbf {T}_{ij} \varvec{\mu }_j} \right] , \end{aligned}$$

where $\alpha _i$ is the atomistic polarizability of atom *i*, $\varvec{r}_i = \{x_i, y_i, z_i\}$ is the position of the atom *i*, and $\varvec{T}_{ij}$ is the dipole field tensor given by Ref.^36^:

6 $$\begin{aligned} \mathbf {T}_{ij} = {\left\{ \begin{array}{ll} \frac{1}{\Delta r^3} \mathbf {I} - \frac{3}{\Delta r^5} \begin{bmatrix} \Delta x^2 &{} \Delta x \Delta y &{} \Delta x \Delta z \\ \Delta x \Delta y &{} \Delta y^2 &{} \Delta y \Delta z \\ \Delta x \Delta z &{} \Delta y \Delta z &{} \Delta z^2 \end{bmatrix},&{} \text {if } \Delta r>s\\ \frac{4v^3-3v^4}{\Delta r^3} \mathbf {I} - \frac{3v^4}{\Delta r^5} \begin{bmatrix} \Delta x^2 &{} \Delta x \Delta y &{} \Delta x \Delta z \\ \Delta x \Delta y &{} \Delta y^2 &{} \Delta y \Delta z \\ \Delta x \Delta z &{} \Delta y \Delta z &{} \Delta z^2\end{bmatrix},&\text {if } \Delta r<s. \end{array}\right. } \end{aligned}$$

Here $\mathbf {I}$ is the identity matrix, $\{\Delta x, \Delta y, \Delta z\} = \{x_i-x_j, y_i-y_j, z_i-z_j\}$, $\Delta r \smash {= \sqrt{\Delta x^2 + \Delta y^2 + \Delta z^2}}$ is the distance between atoms *i* and *j*, $v=\Delta r/s$, and $s = 1.662(\alpha _i\alpha _j)^{1/6}$. It includes a filter function to “smear” out the dipoles to avoid a polarization catastrophe developed by Thole el al.^36^, where the magnitude of the dipole interaction can become infinite under certain conditions^37^.

The polarizability vector $\varvec{\alpha }_k$ defines the response of a molecule to a given electric field, where the subscript $\varvec{{k}}$ denotes the direction of the electric field. Given a uniform electric field across the molecule such that $\varvec{E}_{k}(\varvec{r}_{i})=\varvec{E}_{k}$ at all atoms, the polarizability vector can be calculated from the induced dipole moment in Eq. (7) as

7 $$\begin{aligned} {\varvec{\alpha }}_{{k}} = \sum _{i=1}^N\frac{\varvec{\mu }_{i}(\varvec{E}_{{k}})}{||\varvec{E}_{{k}}||}. \end{aligned}$$

The polarizability tensor of the molecule is then given by the combination of the polarizability vectors for three orthogonal electric field directions ${\varvec{x}}$, ${\varvec{y}}$ and, ${\varvec{z}}$:

8 $$\begin{aligned} \varvec{\alpha }_{mol} = \begin{bmatrix} \begin{bmatrix}  \varvec{\alpha }_{{\varvec{x}}} \end{bmatrix} \begin{bmatrix}  \varvec{\alpha }_{{\varvec{y}}} \end{bmatrix} \begin{bmatrix}  \varvec{\alpha }_{{\varvec{z}}} \end{bmatrix} \end{bmatrix}. \end{aligned}$$

The average polarizability is^28^

9 $$\begin{aligned} \bar{\alpha }_{av} = \frac{\lambda _1 + \lambda _2 + \lambda _3}{3}, \end{aligned}$$

where $\lambda _1, \lambda _2$ and $\lambda _3$ are the eigenvalues of $\varvec{\alpha }_{{mol}}$.

## Computational methods

To calculate the polarizability from the spatial coordinates of each atom in the molecule, we evaluate the induced dipole moments of every atom using Eq. (5), which can be arranged as:

10 $$\begin{aligned} \widetilde{\mathbf {A}} \widetilde{\varvec{\mu }} = \widetilde{\mathbf {E}}, \end{aligned}$$

where

11 $$\begin{aligned} \widetilde{\mathbf {A}}= \begin{bmatrix} \varvec{\alpha }_1^{-1} &{} \mathbf {T}_{12} &{} \dots &{} \mathbf {T}_{1N} \\ \mathbf {T}_{21} &{} \varvec{\alpha }_2^{-1} &{} \dots &{} \mathbf {T}_{2N} \\ \vdots &{} \vdots &{} \ddots &{} \vdots \\ \mathbf {T}_{N1} &{} \mathbf {T}_{N2} &{} \dots &{} \varvec{\alpha }_{N}^{-1} \end{bmatrix} \end{aligned}$$

is a $3N\times 3N$ matrix containing the polarizability tensors $\mathbf {T}_{ij}$ of each pair of atoms on its off-diagonals, and on its diagonals the atomic polarizability matrices for each atom *i*, given by

12 $$\begin{aligned} \varvec{\alpha }_i^{-1} = \begin{bmatrix} \alpha _i^{-1} &{} 0 &{} 0\\ 0 &{} \alpha _i^{-1} &{} 0\\ 0 &{} 0 &{} \alpha _i^{-1} \end{bmatrix}. \end{aligned}$$

$\widetilde{\mathbf {E}}$ is a $1\times 3N$ vector containing the electric field at each atom and $\widetilde{\varvec{\mu }}$ is a $1\times 3N$ vector containing the induced dipole moment of each atom; i.e.

13 $$\begin{aligned} \widetilde{\varvec{\mu }}= \begin{bmatrix} \varvec{\mu }_{1}\\ \varvec{\mu }_{2}\\ \vdots \\ \varvec{\mu }_{N} \end{bmatrix}, \quad \widetilde{\mathbf {E}}= \begin{bmatrix} \varvec{E}_{1}\\ \varvec{E}_{2}\\ \vdots \\ \varvec{E}_{N} \end{bmatrix}, \end{aligned}$$

where $\varvec{E}_{i} = [E_{i,x}, E_{i,y}, E_{i,z} ]^\mathrm{T}$ and $\varvec{\mu }_{i} = [\mu _{i,x}, \mu _{i,y}, \mu _{i,z} ]^\mathrm{T}$. As can be seen from Eq. (10), $\widetilde{\varvec{\mu }}$ can be found from the inverse of $\widetilde{\mathbf {A}}$ as $\widetilde{\varvec{\mu }} = \widetilde{\mathbf {A}}^{-1} \widetilde{\mathbf {E}}$. Assuming a uniform electric field, the polarizability vector of the molecule can then be calculated using Eq.( 7), and from this the polarizability tensor $\varvec{\alpha }_{mol}$ and average polarizability $\bar{\alpha }_{av}$ can be calculated using Eqs. (8)  and (9), respectively.

### Testing with small molecules

To verify the accuracy of the model, it was tested on small molecules with known experimental bulk average polarizabilities. The values for the atomic polarizability $\alpha _i$ used in this paper are displayed in Table 1, and the *x*, *y*,  and *z* co-ordinates of each atom in a given molecule were obtained from Ref.^39^. Henceforth, we assume a uniform electric field $\varvec{E}_{k}$ across the molecule. However, the induced dipole moment, and therefore the scattered power, could be calculated for any applied field, such as the highly nonuniform fields typical of plasmonic biosensors^9,10^.

**Table 1:** Atomic polarizabilities used in calculations.

Atom	Polarizability ($\text {\AA }^3$)
H	$0.514^{\mathrm{a}}$
C	$1.405^{\mathrm{a}}$
N	$1.105^{\mathrm{a}}$
O	$0.862^{\mathrm{a}}$
S	$2.900^{\mathrm{b}}$
P	$3.630^{\mathrm{b}}$

$^{\mathrm{a}}$Values from Ref.^36^, $\lambda = 589.3$ nm.

$^{\mathrm{b}}$Values from Ref.^38^.

Table 2 compares the average molecular polarizability calculated here (column C) to those reported by Applequist et al.^28^ (column A), Thole^36^ (column T), and experimental values^28^ (column E). Applequist et al. reports values for average polarizability that differ from calculated values by up to 22 % (for $\hbox {H}_2\hbox {O}$). We attribute these discrepancies to the polarization catastrophe discussed earlier. Indeed, we found this catastrophe to become increasingly problematic as the molecular size became larger, so that it was not possible to obtain accurate results without Thole’s correction. Our model, as described so far, is identical to the one used by Thole, and yielded very similar results (within 1 %) with minor differences attributed to the use of slightly different molecular coordinates. As shown in the table, both sets of results agree well with the experimental average polarizability, with a maximum deviation of 4 %.

**Table 2:** Experimental versus calculated polarizabilities for various small molecules: Experimental (E), Applequist et al. (A), Thole (T), Calculated (C). $\lambda = 589.3$ nm.

Compound	Average polarizability($\text {\AA }^3$)			
	$\hbox {E}^{\mathrm{a}}$	$\hbox {A}^{\mathrm{a}}$	$\hbox {T}^{\mathrm{b}}$	C
Methane ($\text {CH}_4$)	2.62	2.58	2.55	2.55
Ethane ($\text {C}_2\text {H}_6$)	4.48	4.47	4.46	4.43
Propane ($\text {C}_3\text {H}_8$)	6.38	6.58	6.29	6.32
Cyclohexane ($\text {C}_6\text {H}_{12}$)	11.00	10.95	10.95	11.02
Formaldehyde ($\text {CH}_2\text {O}$)	2.45	2.46	2.54	2.51
Dimethyl ether ($\text {C}_2\text {H}_6\text {O}$)	5.24	5.22	5.24	5.26
Acetone (($\text {CH}_3)_2\text {CO}$)	6.39	6.44	6.32	6.34
Water ($\text {H}_2\text {O}$)	1.49	1.12	1.44	1.43

Values from Ref.^36^ Values from Ref.^28^.

### Scaling up to larger molecules

Motor molecules and other proteins are much larger than the molecules computed in “Testing with small molecules ” section (for example, the motor molecule ATPase has $N =$ 39,000 atoms). As there are $9N^2$ elements in $\widetilde{\mathbf {A}}$, computer memory constraints fast become a problem when trying to compile $\widetilde{\mathbf {A}}$ for larger molecules. In our case, this has prevented application of the method for $N >$ 17,000.

The matrix $\widetilde{\mathbf {A}}$ contains the polarizability tensor of each atom as well as the dipole field tensors for each dipole formed by two atoms (Eq. 11). The magnitude of the dipole field tensor scales as $1/\Delta r^5$. Therefore, as the distance between pairs of atoms increases their contribution to the overall polarizability falls rapidly and can eventually be neglected (set to zero). This leads to a sparse matrix and a dramatic reduction in the total number of matrix elements that need to be stored. We introduce a threshold radius $\Delta r_t$, and set $\mathbf {T}_{ij} = 0$ for all elements in $\widetilde{\mathbf {A}}$ for which $\Delta r > \Delta r_t$.

**Figure 3:**
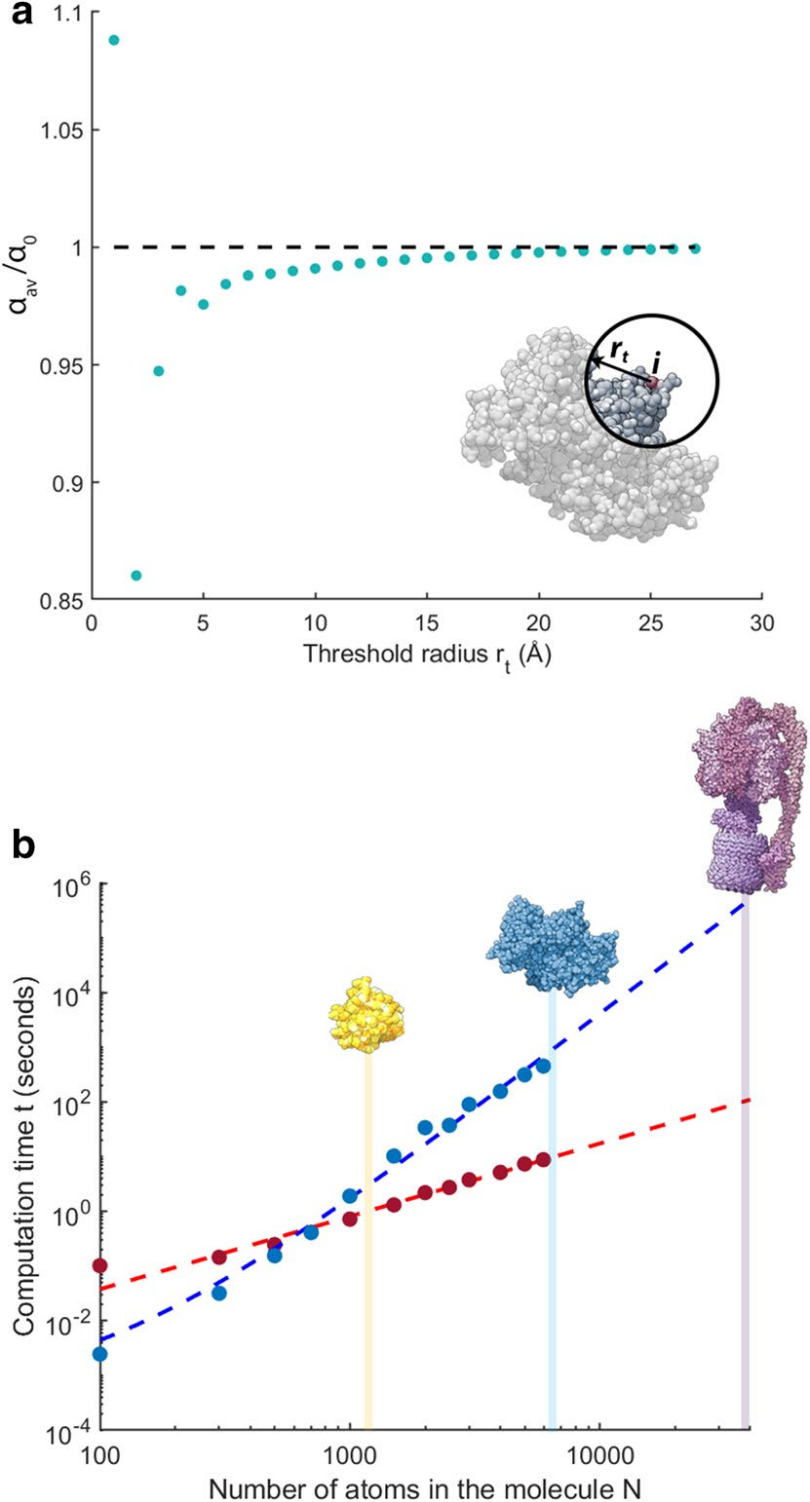
(**a**) Threshold radius versus average molecular polarizability ($\alpha _{av}$). $\alpha _0$ is the average polarizability of BSA [PDBID: 3V03]^40^ with no threshold radius applied. (**b**) Comparison of the time taken to calculate $\widetilde{\varvec{\mu }}$ by inverting $\widetilde{\mathbf {A}}$ (in blue) and by using an iterative solver (in red). Sizes of various proteins (Photoactive yellow protein [PDBID: 3VE4], BSA, and ATPase [PDBID: 6FKI]).

To test at what threshold radius matrix elements can be neglected, the polarizability determined for a single protein with and without a threshold radius is compared in Fig. 3a. Bovine Serum Albumin (BSA) is used for this comparison. BSA is commonly used in label-free single molecule experiments^4,23^. We derive its molecular structure from the protein data bank file [PDBID: 3V03]^40^ which contains 8828 atoms, well below the size at which memory errors become a problem ($N =$ 17,000). In our model, we reduce the effective atom number to 5944 by removing hetero atoms and using a united atom model. Hetero atoms are atoms that are not part of the protein, such as water, buffer salt ions, ligands and crystallization media. These atoms were removed because they are due to the crystallisation of the BSA molecule for X-ray diffraction measurements, and not representative of the BSA molecule under regular conditions. United atom models represent the molecule atomistically, however aliphatic hydrogen atoms that are covalently bonded to larger atoms are grouped with the larger atoms. Aliphatic hydrogen atoms have much less charge than the carbon atoms with which they are bonded, removing these hydrogen atoms and making the carbon atoms slightly larger reduces the time required to simulate the molecule. This has negligible effect on the results of the simulation^41^.

As shown in Fig. 3a, at a threshold radius of 1 $\text {\AA }$, the thresholded calculation overestimates the polarizability of BSA by approximately 10 %. This is because all atoms are separated by greater distances than 1 $\text {\AA }$, and so all atom-atom dipoles are excluded from the molecular dipole. The resulting molecular polarizability is then just the sum of the atomic polarizabilities of each atom ($\alpha _0$). At thresholds close to but above 2 $\text {\AA }$, the polarizability is underestimated by a similar margin. This indicates that including only interactions from closely separated atoms causes a decrease in the estimated polarizability. As atomic interactions with greater separations are included, the polarizability asymptotes towards the value obtained with no threshold radius applied. For instance, for a threshold radius of $\Delta r_t = 30 \text {\AA }$, the polarizability is within 99 % of the threshold-less value. Henceforth, we use this $30\,\text {\AA }$ threshold radius in our calculations. We found that this significantly reduced memory requirements, allowing calculations for molecules as large as $N =$ 110,000.

We note that the calculations in Fig. 3a shed some light on when it may be appropriate to use a bulk model or a model based on a collection of sub-domains to calculate the polarizability (e.g. see Ref.^25^). If the effective radii of each sub-domain is greater than one nanometer, then it can be expected that interactions between the sub-domains will not affect the polarizability significantly. For smaller, closely connected sub-domains, the interactions will be significant, and they should be explicitly considered in any model used to determine the polarizability. For instance, an aggregate of several BSA molecules. Since each molecule is roughly 3 nm in radius, after performing an atomistic calculation of the polarizability of one BSA molecule, the polarizability of the aggregate could be expected to be estimated by treating it as a compound system of non-interacting parts, each with the same polarizability.

Equation (10) can be solved for $\widetilde{\varvec{\mu }}$ by inverting $\widetilde{\mathbf {A}}$. However, even with a sparse matrix we found that the time required to perform this operation increased rapidly with molecule size, making the cost of this approach prohibitive even for relatively small proteins. This is shown quantitatively in Fig. 3b. The computational time scales as $N^{3.5}$ for molecules with more than 500 atoms. To address this limitation, the use of an iterative solver, the MATLAB minimum residual method (minres), was examined. While this solver iterates towards a solution to the matrix inverse problem, rather than solving directly, we find that it offers sufficient accuracy with greatly reduced time overhead when dealing with large molecules. For small molecules ($N <$ 700), directly inverting $\widetilde{\mathbf {A}}$ is faster. However, the iterative method scales as $N^{1.3}$ (see Fig. 3b) allowing a dramatic speed up for large molecules. For instance, it was possible to calculate $\widetilde{\varvec{\mu }}$ for ATPase ($N =$ 39,000) in 106 seconds, compared to 130 hours using a direct matrix inverse.

A caveat for the iterative method is that it requires a tolerance for accuracy to be set before the calculation. The solver will continue to approximate the result over a given number of iterations until the residual of the result is below this tolerance level. Increasing the tolerance can decrease run time but it can also decrease accuracy of the result. In this paper, we use a tolerance of $10^{-6}$, which is the default tolerance for the minres function in MATLAB.

### Testing with bulk materials

Combining the sparse matrix and iterative solver discussed in “Scaling up to larger molecules” section, we were able to extend the polarizability calculations to relatively large molecules, and to test the method with bulk materials that have well-known refractive indices. Polarizability is related to the refractive index via the Lorentz-Lorenz relation:

14 $$\begin{aligned} \frac{n^2-1}{n^2+2} = \frac{4\pi }{3}\rho \bar{\alpha }_{av}, \end{aligned}$$

where *n* is the refractive index and $\rho$ is the atomic density per cubic metre^42^.

The polarizability of two bulk materials, diamond and water, was calculated. For diamond, we used a crystal lattice constant of $a = 3.567\,\text {\AA }$ to generate a set of atomic coordinates^44^. For water, we used a snapshot taken from a molecular dynamics simulation of liquid water at 273 K at atmospheric pressure. The water was described using the TIP5P model^45^. Table 3 shows the refractive indices calculated based on these structural models using our approach versus experimental values. The calculated refractive indices for diamond and water are within 2 % and 0.2 % of the experimental values, respectively. This close agreement provides some confidence in the ability of the model to accurately calculate the molecular polarizability of biomolecules.

**Table 3:** Polarizability of bulk materials.

Material	$\bar{\alpha }_{av}$($\text {\AA }^3$)	Calculated *n*	Experimental *n*
Diamond	4030	2.458	$2.417^{\mathrm{a}}$
$\hbox {H}_2\hbox {O}$	3944	1.3299	1.3325

$^{\mathrm{a}}$Values from Ref.^43^. Experimental refractive indices are at a wavelength of $\lambda = 589.3\,$nm.

### The effect of molecular geometry on polarizability

**Figure 4:**
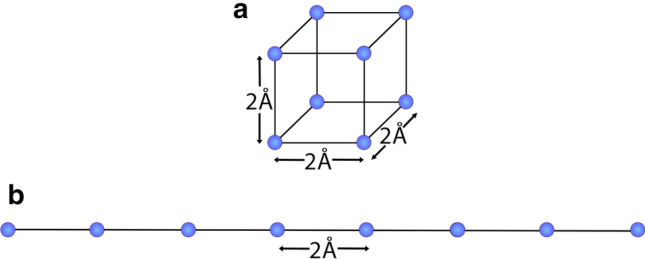
The shape of a molecule affects its polarizability. For example, we find that a cube of 8 carbon atoms separated by 2 $\text {\AA }$ has an average polarizability of $12\,\text {\AA }^3$ (**a**) and a line of the same 8 atoms separated by 2 $\text {\AA }$ has an average polarizability of $20\,\text {\AA }^3$ (**b**).

As illustrated by Eq. (14), bulk models of polarizability average-out the microscopic configuration of atoms, and rather employ the bulk properties of the material or molecule. Of course, the polarizability does depend on how the atoms are arranged, so that changing the shape of a molecule will influence the average polarizability.

To illustrate this in a simple example, we use our method to predict the polarizability of two hypothetical molecules constructed purely from carbon atoms. In the first, shown in Fig. 4a, eight carbon atoms are arranged in a cube. In the second (Fig. 4b), they are arranged in a line, with the same 2 $\text {\AA }$ separation between nearest-neighbour atoms.

Even though both structures contain the same number of atoms and nearest-neighbour separation, the cube has an average polarizability of $12\,\text {\AA }^3$ and the line of atoms has an average polarizability of 20$\,\text {\AA }^3$. When applying an electric field $\varvec{E}_{k}$, where k is the axis parallel to the length of the elongated molecule, the magnitude of the polarizability vector $||\varvec{\alpha }_{\varvec{ex,k}}||$ is $12\,\text {\AA }^3$ for the cube-shaped molecule and $43\,\text {\AA }^3$ for the elongated molecule. This illustrates the significantly impact that reorganization of a molecule can have on its polarizability.

## Results

### Polarizability of Bovine Serum Albumin

As a first application of our method to larger molecules, we considered the case of a single BSA molecule [PDBID: 3V03]^40^. Our approach yields an average molecular polarizability $\bar{\alpha }_{av}$ of $5860\,\text {\AA }^3$, averaged over a 54 ns simulation with 1080 time data points. Two replicate molecular dynamics simulations yielded the same value for $\bar{\alpha }_{av}$ within 0.2 %. These replicate simulations used the same Protein Data Bank (PDB) file for BSA [PDBID: 3V03]^40^ but had different starting velocities generated from a Maxwell-Boltzmann distribution at 300 K.

For comparison, Vollmer et al. use a bulk model to calculate an excess polarizability for BSA of $3850\,\text {\AA }^3$^23^ which, accounting for the polarizability of the displaced water, corresponds to a molecular polarizability of around $7800\,\text {\AA }^3$. This is reasonably close to the result of our calculation. The good agreement of our model with known results for bulk materials is indicative that the discrepancy may arise from the use of a bulk model in Ref.^23^.

### Conformational changes due to thermal fluctuations

**Figure 5:**
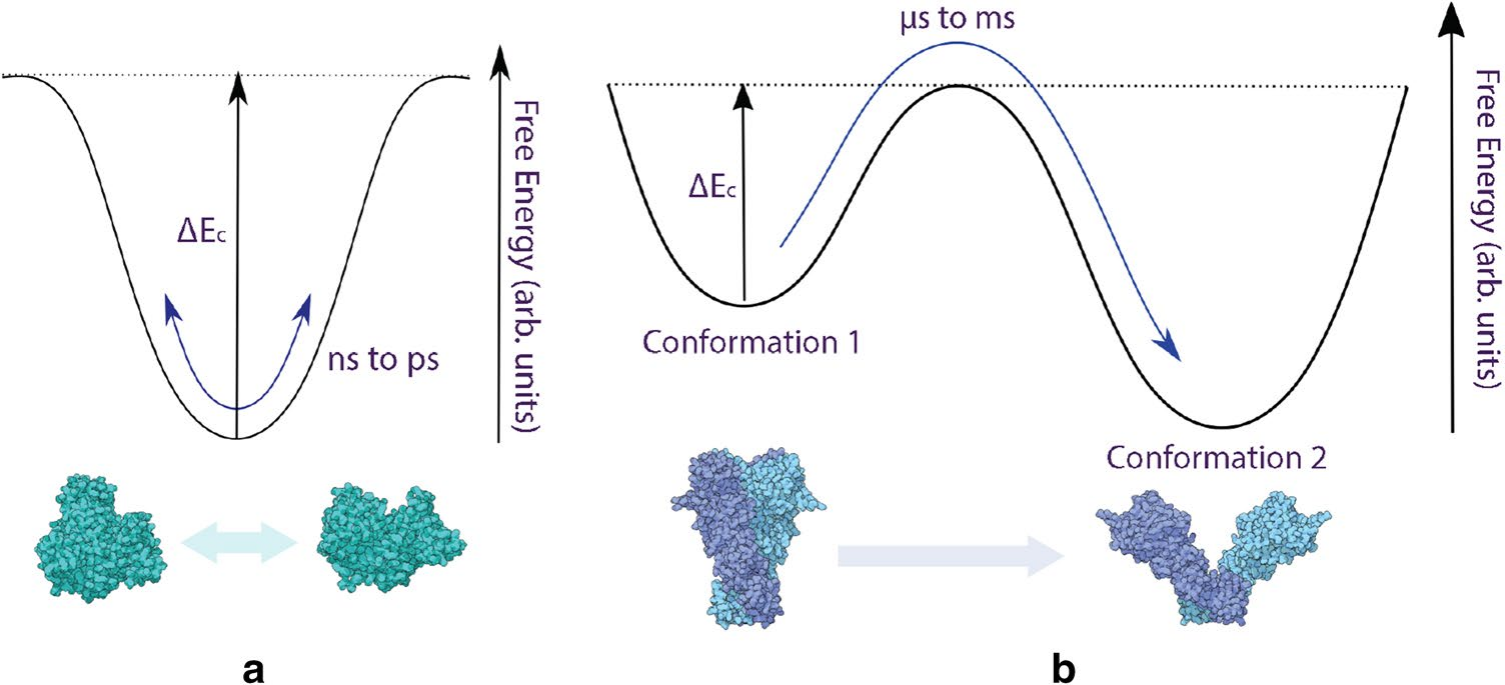
(**a**) Fluctuations in polarizability on a picosecond-nanosecond time scale are typically due to thermal fluctuations of a single molecular conformation^46^, [PDBID: 3V03]^40^, illustrative purposes only. (**b**) Free energy diagram of a conformational change of a molecule, which typically occurs over a time scale of microseconds to milliseconds^46^, [PDBID: 2CG9, 2IOQ], illustrative purposes only.

So far, we have treated each biomolecule as a rigid structure, that is, a collection of atoms with fixed coordinates. However, in reality proteins are highly flexible. They reside in multiple local minima at room temperature and fluctuate around these local minima on a wide variety of timescales. This is illustrated diagrammatically^47^ in Fig. 5. Figure 5a represents motion around a local minima while Fig. 5b represents two alternative quasi-equilibrium conformational states separated by an energy barrier. An attractive aspect of the methodology we have developed is that it opens the possibility to probe structural transitions in single molecules on the timescales accessible to molecular dynamics simulation techniques. In principle this makes it possible to directly relate temporal fluctuations in the polarizability measured experimentally to changes in underlying molecular configuration as probed using atomistic simulations. As an illustration of this potential we have compared temporal measurements of the polarizability of a single molecule of BSA freely rotating in aqueous solution at 273 K to results from a series of 54 ns molecular dynamics simulations of BSA described using the GROMOS 54A7 force field^48–50^ in TIP5P water^45^. See supplementary for more information.

**Figure 6:**
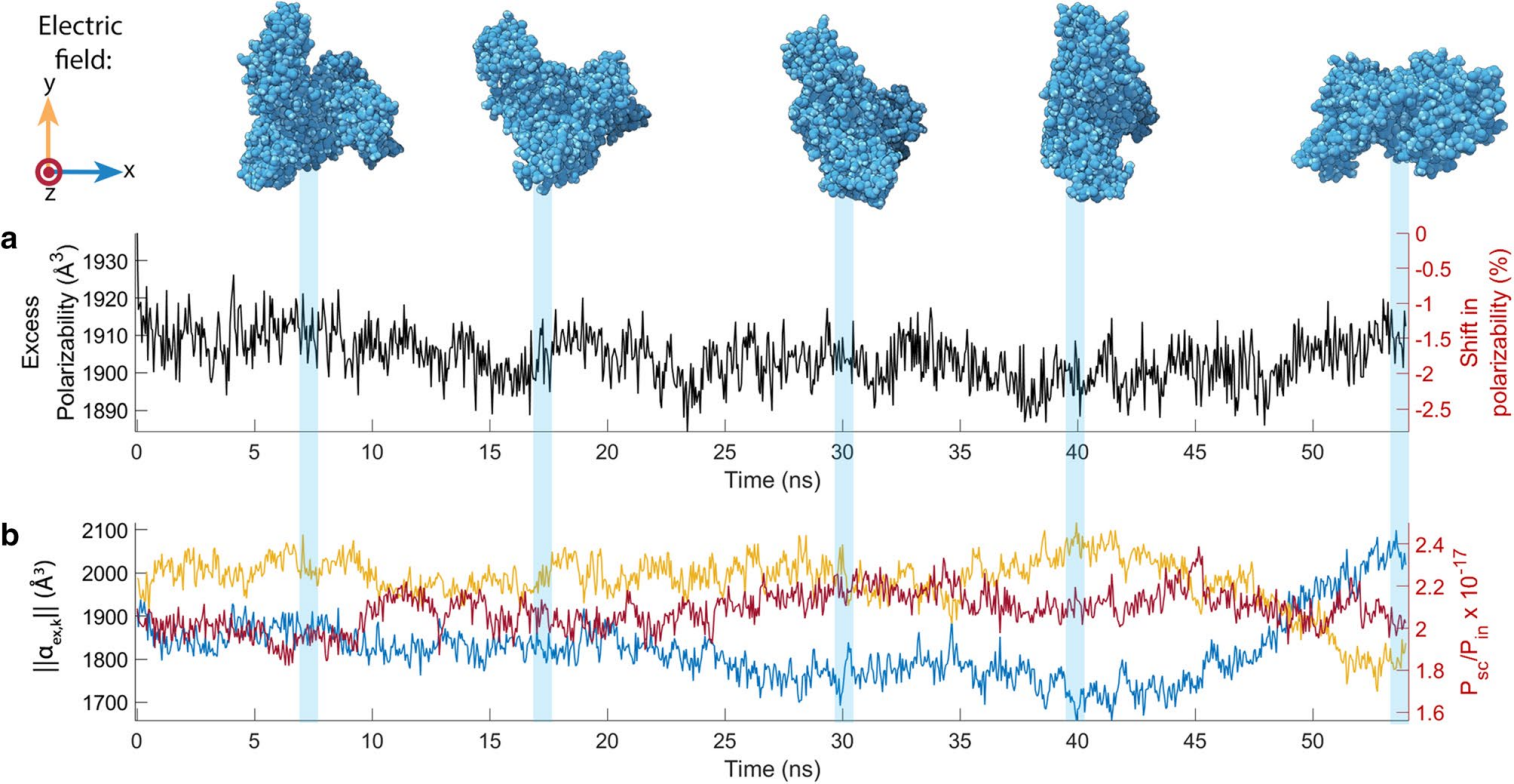
(**a**) Average excess polarizability fluctuations of BSA over 54 nanoseconds. (**b**) Magnitude of the polarization vector of BSA with polarized input light (in red, yellow and blue), with arrows indicating the optical electric field direction. [PDBID: 3V03]^40^.

Figure 6a plots the change in the average excess polarizability $\bar{\alpha }_{ex}$ of BSA at 50 ps intervals from a typical simulation. The average polarizability varies by around 2.5 % over the simulation. The two replicate molecular dynamics simulations for BSA yielded similar variation of the average polarizability over the same time period (3 % and 2.5 %). Note that since the average polarizability is the average of the polarization vectors obtained when applying an electric field in each of the *x*, *y* and *z* directions, it is unaffected by the orientation of the molecule. It is only sensitive to variations in conformation. In this case the conformations sampled correspond to a system sampling a local minmima or in a quasi-steady state (illustrated in Fig. 5a).

Under illumination from a spatially uniform light field with optical polarisation *k*, the scattered optical power from the molecule (Eq. 1) is determined by the magnitude of the excess polarizability vector, defined as $||\varvec{\alpha }_{\varvec{ex,k}}|| = ||\varvec{\alpha }_{\varvec{k}}|| - \bar{\alpha }_{\varvec{m}}$. Fig. 6b plots this magnitude for BSA for three orthogonal optical polarizations ($k = x,y,$ and *z*). From this value, we can directly infer the scattered power for each conformation (right axis). The polarizability vector (as defined in Eq. 7) depends on the direction of the electric field relative to the molecule and is sensitive to the rotation of the molecule as well as to any deformations. With an applied electric field in the *x*-direction, the magnitude of the excess polarizability vector fluctuated by around 27 % over the course of the 54 ns simulation. For electric fields applied in the *y*- and *z*-direction, it changed by 19 % and 16 % respectively. That the change in ||$\varvec{\alpha }_{\varvec{ex,k}}$|| is up to 10 times greater than change in average excess polarizability indicates that rigid rotation of the BSA molecule dominates over thermally driven deformations.

Fluctuations in light scattering due to thermally-driven changes in the excess polarizability vector are a potential source of error in single-molecule sensing techniques such as mass photometry^6^. Our BSA simulations allow us to roughly estimate the magnitude of such errors. Taking, for example, an applied electric field in the *x*-direction, and assuming that the fluctuations in the excess polarizability are Gaussian, we find that the standard-error in the excess polarizability vector is around 0.13%. We conclude, therefore, that thermally-driven fluctuations in the excess polarizability can be expected to be a negligible source of noise, even for a 50 ns measurement. We note that the standard-error typically scales as the inverse-square-root of measurement time, and should therefore be negligible in realistic scenarios, where the measurement duration is on the order of milliseconds^4^. This is consistent with observations in Ref.^6^.

### Distinguishing between different quasi-equilibrium conformational states

**Figure 7:**
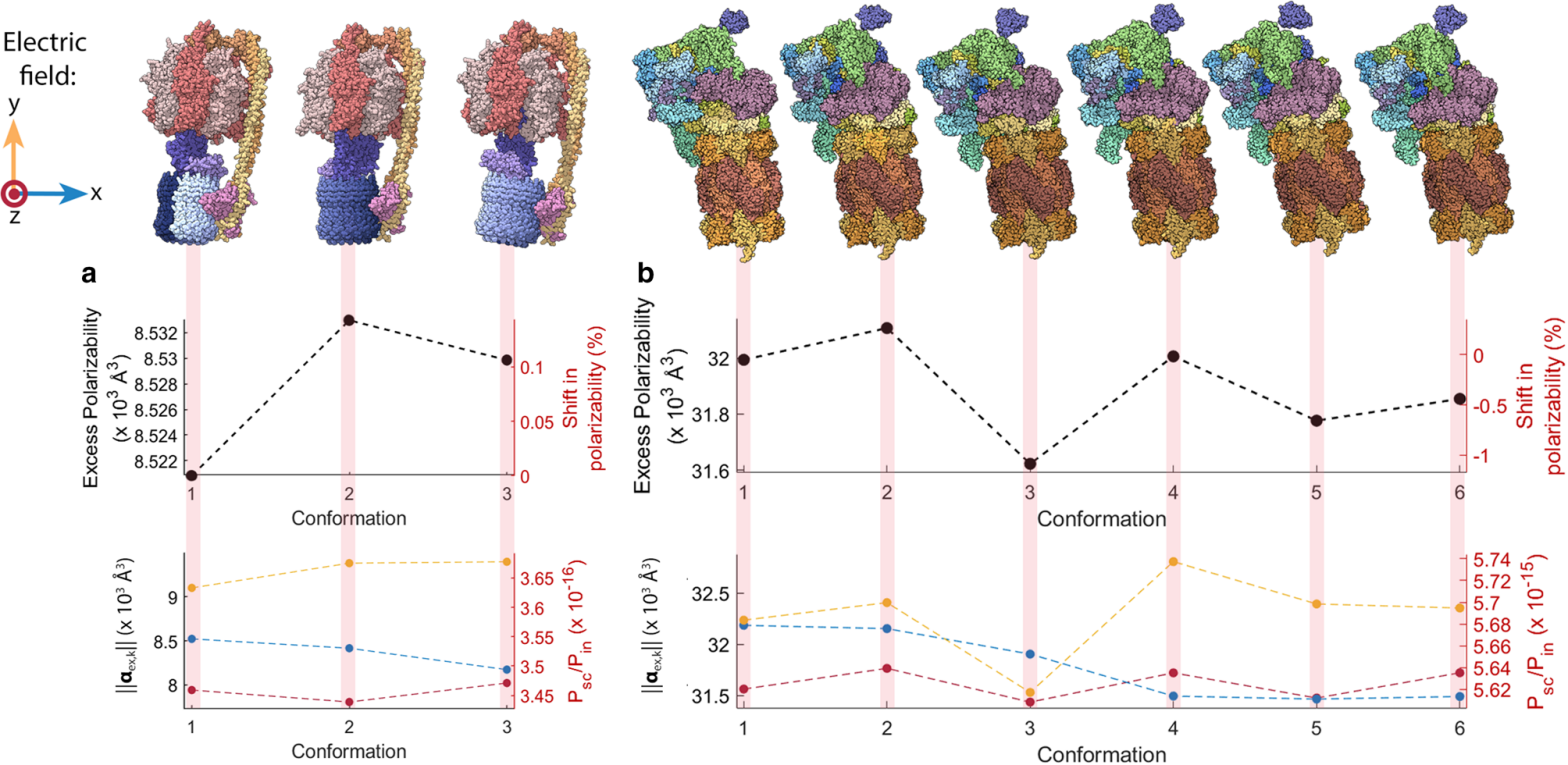
Average excess polarizability *(top)* and the magnitude of the polarizability vector for three orthogonal electric field vectors ($k = x,y,$ and $z$) *(bottom)* for different conformations of (**a**) chloroplast $\text {F}_1\text {F}_0$ ATP synthase [PDBID: 6FKF, 6FKH, and 6FKI] and (**b**) 26S proteasome [PDBID: 6FVT, 6FVU, 6FVV, 6FVW, 6FVX, and 6FVY].

The measurements and simulations in Fig. 6 represent a system that is close to a single quasi-equilibrium state. Transitions between alternative quasi-equilibrium states (as illustrated in Fig. 5b^46^) are key to the function of many proteins. Coordinates for thermally accessible alternative conformations of a wide range of proteins are available from the Protein Data Bank^51^. We have used our methodology to assess whether transitions between these alternative conformations might be detected experimentally based on predicted changes in polarizability. For this, two molecules have been considered: Chloroplast F1F0 ATPase [PDBID: 6FKF, 6FKH, and 6FKI]^52^ (Fig. 7a) and 26S Proteasome [PDBID: 6FVT, 6FVU, 6FVV, 6FVW, 6FVX, and 6FVY]^53^ (Fig. 7b). We note that this approach neglects thermally driven deformations and reorientations of the molecules. These motions will modulate the optical scattering, and therefore introduce additional noise when distinguishing between conformations. Our analysis for BSA in the previous section suggests that this noise may be negligible. The same sort of molecular modelling as was performed in that section could be employed to test this.

Chloroplast $\text {F}_1\text {F}_0$ ATPase is a rotary enzyme complex that uses a proton gradient across the thylakoid membrane to drive the production of ATP. It has a mass of 597 kDa, and consists of roughly 39,000 atoms. The enzyme complex is believed to adopt three distinct conformational states during each rotation. The spatial coordinates of the chloroplast $\text {F}_1\text {F}_0$ ATPase atoms in each of these three conformational states have been inferred from cryo-EM data^52^ and are illustrated in Fig. 7. The rotor consists of c subunits (coloured in white-blue gradient), and central stalk subunits ($\gamma$ in purple and $\varepsilon$ in lilac). Between conformation 1 and 2 the rotor turns $112^{\circ }$, and between conformation 2 and 3 it rotates $103^{\circ }$. The remaining $145^{\circ }$  is the angle between the conformation 3 and 1^52^. The rotor of labelled ATPase completes one full rotation in 2.2 ms^54^.

Figure 7a (*top)* shows a plot of the average excess polarizability of chloroplast $\text {F}_1\text {F}_0$ ATPase in each of its three conformational states. The average excess polarizability increases by 0.14 % from conformation 1 to 2, and decreases by 0.04 % from conformation 2 to 3. The average polarizability then decreases by 0.1 % between conformation 3 and 1. Since the average excess polarizability is insensitive to the orientation of the molecule, these differences stem from changes in conformation.

The magnitude of the polarizability vector ||$\varvec{\alpha }_{\varvec{ex,k}}$|| of each ATPase conformation is plotted in Fig. 7a (*bottom)* under three applied optical electric field directions ($k = x,y,$ and $z$). The protein has been orientated such that the *x*, *y* and *z* axes correspond to those in the protein’s PDB coordinate file. With the electric field applied along the *x*-axis, the polarizability decreases by 1.2 % between conformations 1 and 2, and decreases a further 2.9 % between conformation 2 and 3. It then increases by 4.1 % between conformation 3 and 1. For an electric field along the *y*-axis, the magnitude of the polarizability increases by 3.1 % between conformation 1 and 2, and a further 0.2 % between conformation 2 and 3, decreasing by 3.3 % between conformation 3 and 1. For a *z*-axis electric field, the polarizability magnitude first decreases by 1.7 % between conformation 1 and 2, then increases by 2.7 % between conformation 2, and decreases by 1 % between conformation 3 and 1.

The changes in polarizability vector magnitude in Fig. 7 likely arise due to large scale reorganisation that occurs between conformations. As illustrated in Fig. 4, such reorganisation can be expected to affect the polarizability of a molecule. The primary such change for ATPase is the rotation of the central stalk. Note, the $\varepsilon$ subunit (coloured lilac in Fig. 7a) moves around the outside of the central stalk as it rotates. The ATPase molecule also tilts at different angles relative to the *y*-axis in each conformational state. For example, conformations 2 and 3 are tilted by $10.2^{\circ }$  and $11.5^{\circ }$  relative to conformation 1, respectively.

We find that the changes in the magnitude of the polarizability vector between conformations are about an order of magnitude greater than the changes in average polarizability. For instance, the average polarizability increases by 0.14 % between conformation 1 and 2, compared to 3.2 % for the magnitude of the *y*-axis polarizability vector. Changes in average polarizability are likely to predominantly result from a change in the average separation of atoms, and therefore to correspond roughly to a change in the density of the molecule.

Figure 7b shows magnitude of the polarizability vector and the average polarizability calculated for 26S proteasome. Based on cryo-EM data for 26S Proteasome (mass 1583 kDa, approximately 110,000 atoms) a series of six conformational states or structural classes have been identified (s1–s6)^53^. Conformational states are dominated by the six ATPase subunits, which undergo concerted cycles of ATP binding, hydrolysis and release^53^, together with the gate or cap which is closed in conformations s1,s2, and s3 and open in conformations s4,s5, and s6^53^.

Figure 7b (*top*) plots the average polarizability predicted for the six major conformational states of 26S proteasome. The average polarizability of proteasome varies between 31,600 $\text {\AA }$ (conformation 3) and 32,100 $\text {\AA }$ (conformation 2). This is a change of approximately 1 % between conformations, ten times greater than that of ATPase.

Figure 7b (*bottom*) shows plots of the magnitude of the polarizability vector when an *x*, *y* or *z* aligned optical field is applied for the six conformational states of 26S proteasome examined. As with ATPase, even though sub-units experience rotation, the entire proteasome molecule does not rigidly rotate more than a few degrees relative to the applied electric field between conformations. This indicates that rigid rotation plays an insignificant role in the changes observed in Fig. 7b.

For an electric field aligned along the *x*-direction, the magnitude of the polarizability vector is 32,000 $\text {\AA }^3$ or above for s1, s2, and s3 (gate closed), but decreases to 31,500 $\text {\AA }^3$ for s4, s5, and s6 (gate open). This suggests that the opening and closing of the gate could potentially be resolved from changes in the scattering cross-section of the molecule. This would require that the 26S proteasome is bound to a surface to prevent free rotation that would result in an averagiung of the polarizability vectors for the three optical field alignments. With a *y*-axis electric field, the magnitude of the polarizability vector dips notably for conformation s3, a dip that is also observed in the average polarizability. When applying a *z*-axis electric field, the magnitude of the polarizability vector changes the least, with maxima in the s2, s4 and s6 states.

26S Proteasome is composed of thirty-three unique proteins that reorganise between conformations. Thus it is difficult to infer what exact structural differences are causing the changes in polarizability predicted by our model. Unlike ATPase, however, the changes in average polarizability between 26S proteasome conformations are on the same order of magnitude as the changes in the magnitude of the polarizability vector. This suggests that changes in inter-atom separation play a significant role in the differences in polarization between conformations.

### Detectability via optical scattering

Molecular conformational changes have been successfully detected using optoplasmonic sensors^25^. In this approach the high spatial gradients that exist in the electric field of a plasmonic resonator are used. Motion shifts a significant fraction of the molecule out of the plasmonic hot spot, resulting in a reduction in scattering. It is interesting to ask whether the polarizability changes predicted here could also provide a means to observe changes in molecular conformation. That is, whether the changes in optical scattering due to alterations in molecular conformation could be resolvable using state-of-the-art single-molecule biosensors such as those in Ref.^4–6^ even in the case that their electric field was uniform.

Let us take the specific case of Ref.^4^, where the molecule is illuminated by a tightly focused incident field and the scattered light is collected in an optical nanofibre. In such an arrangement the total scattered power is related to the polarizability through Eq. (1)^55^. Replacing the term $||\varvec{\mu }_{ex}||$ in this equation with its equivalent $||\varvec{\alpha }_{ex,k}|| \times ||\varvec{E}_{k}||$ and converting the magnitude of the electric field into scattered optical power gives

15 $$\begin{aligned} \langle P_{sc} \rangle = \frac{k^4}{24\pi ^2{{\varepsilon }_m}^2w^2} ||\varvec{\alpha }_{ex,k}||^2 \langle P_{in} \rangle , \end{aligned}$$

where *w* and $\langle P_{in} \rangle$ are the width and mean power of the incident field, respectively.

The right axis of Fig. 6b shows the scattered power from a BSA molecule in water undergoing thermal motion at 273 K as a function of time, calculated using Eq. (15) and normalised to the incident optical power. We assume an optical wavelength of $\lambda = 780$ nm, and that the incident field is focused to a waist with width of $w=1\,\upmu$m. As can be seen, this is a factor of around $10^{17}$ lower than the input power, meaning that only around one in $10^{17}$ photons will be scattered by the molecule. The scattered power from each conformation of ATPase and proteasome are shown on the right axes in Fig. 7a, b *(bottom)*. Here, the scattered power is around $10^{16}$ and $10^{15}$ times smaller than the input power, respectively.

**Figure 8:**
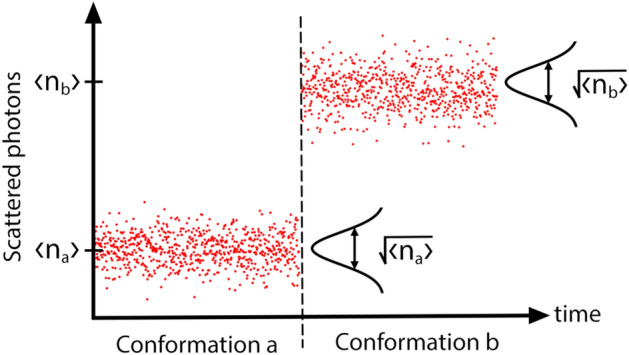
Illustration of the scattered photon flux that might be detected prior to, and after, a conformational change. Here, the random variation in the collected photon number within each period where the conformation is constant can be attributed to optical shot noise, while the discrete jump in the mean scattered photon number can be attributed to the change in polarizability due to the conformational change. Here $\langle n_a \rangle$ and $\langle n_b \rangle$ are the mean scattered photon flux over the measurement time in conformations *a* and *b*, respectively. The difference between mean scattered photon number from states *a* and *b* must be greater than the noise for the change to be resolvable.

Let us consider an experiment that counts the number of scattered photons over some integration period (the temporal resolution of the measurement), and records this photon count as a function of time. To determine whether a conformational change has occurred at a particular time, one can compare the photon counts in the integration periods immediately before and after this time. We denote these photon counts $n_a$ and $n_b$, respectively. This approach is illustrated in Fig. 8. As shown, to resolve a conformational change the mean change in photon count between the two conformation must be larger than the combined statistical fluctuations in the photon counts of the two conformation involved; i.e. the absolute value of the mean of the measured signal $\langle i \rangle = \langle n_b \rangle - \langle n_a \rangle$ must be larger than its standard deviation $\sqrt{\langle i^2 \rangle - \langle i \rangle ^2}$.

A number of noise sources can contribute to the statistical fluctuations, including vibrations, electrical noise, and optical shot noise resulting from the quantisation of light^4^. Brownian motion of the molecule could itself cause perturbations in the measured scattered signal which may obscure conformational dynamics. Translational Brownian motion would not be observed in sensors with a uniform electric field as used in our model. It may be detected in a sensor with a non-uniform electric field as the molecule moves in and out of the detection hotspot, but in this case can be reduced using optical trapping^11,56^ or surface binding^25^. Rotational Brownian motion is a potential source of signal noise in both uniform and non-uniform electric field biosensors as the molecule will change orientation with respect to the input electric field, thus changing the scattered signal magnitude. However, these rotations generally occur on a timescale of nanoseconds and average out over the measurement time of most biosensors ($>\mu \hbox{s}$)^4,56,57^.

A recent experiment has shown that the conformational dynamics of a trapped molecule can be resolved over perturbations in the scattered signal due to Brownian motion^56^. Supported by this observation, here we consider only optical shot noise which constitutes a fundamental noise limit in the absence of quantum correlations between photons^58–60^. This limit has been reached in both dark field heterodyne microscopy^4^ and interferometric scattering microscopy^61^. Because uncorrelated photons have Poissonian statistics, the variance of the optical shot noise is equal to the mean number of photons that would be collected in the time interval of the measurement^62^. As a consequence the standard deviation is $\sqrt{\langle i^2 \rangle - \langle i \rangle ^2} = \sqrt{ \langle n_b \rangle + \langle n_a \rangle }$. The condition to resolve a change in conformation can then be expressed as

16 $$\begin{aligned} |\langle n_a \rangle - \langle n_b \rangle | > \sqrt{\langle n_a \rangle + \langle n_b \rangle }. \end{aligned}$$

The number of photons scattered by a molecule in an integration time $\tau$ is related to the scattered power by

17 $$\begin{aligned} \langle n \rangle = \tau \langle P_{sc}\rangle \lambda /hc, \end{aligned}$$

where *h* is Planck’s constant and *c* is the speed of light. Using Eqs. (1), (16) and (17), and assuming that all scattered photons are collected, the minimum incident optical field intensity needed to resolve a conformational change using a shot noise limited biosensor can be expressed as

18 $$\begin{aligned} I_{min} = \frac{48cn_m^2\pi h}{k^4\lambda }\frac{||\varvec{\alpha }_{ex,k,a}||^2 \tau _a + ||\varvec{\alpha }_{ex,k,b}||^2 \tau _b}{|\, ||\varvec{\alpha }_{ex,k,a}||^2 \tau _a - ||\varvec{\alpha }_{ex,k,b}||^2 \tau _b|^2}, \end{aligned}$$

where $n_m$ is the refractive index of the medium, and the subscripts “a” and “b” are used to label the before and after conformation, respectively. We assume in the below that the medium is water, with $n_m=1.33$.

While the changes in the polarizability of BSA are continuous as it rotates in a medium, we are able to apply Eq. (18) to roughly estimate the illumination intensity that would be required to resolve its motion. We do this by considering the dynamics of $||\varvec{\alpha }_{ex,x}||$ shown in Fig. 6b over the time period from 40 to 54 ns. Over this time, the alignment of the long-axis of the molecule rotates from pointing roughly along the *y* axis, to roughly along the *x*-axis. There is a corresponding change in $||\varvec{\alpha }_{ex,x}||$ from 1700 to 2100 $\text {\AA }^3$. Using these values, and choosing $\tau _a=\tau _b=10$ ns to approximately account for the available measurement integration time for each orientation, we find from Eq. (18) that an intensity of around $I_{min} \approx 5$ MW$/\upmu$m$^2$ would required to resolve such a rotation.

Considering the case of ATPase, each conformation exists for a time of roughly 0.7 ms^54^, so that it is natural to set the integration times to $\tau _a=\tau _b=0.7$ ms. Using, as an example, the polarizability vectors calculated earlier for conformations 1 and 3 when illuminated with an optical field polarized along the *x*-axis (Fig. 7a *bottom*) and using Eq. (18) we find that $I_{min} = 170$ $\hbox {W}/\upmu \hbox {m}^2$.

For the case of the 26S proteasome, we were unable to source experimental data for the time that the molecule persists in each conformation, though it is known that the molecule reconfigures from conformation s1 through to conformation s3 in around 0.6 s^63^. Based on this, we assume that each conformation persists for 0.3 s, and set an integration time of $\tau _a=\tau _b=0.3$ s. Comparing the polarization vectors for conformations s3 and s4 when illuminated with a *y*-polarized optical field, we find that these conformations could be distinguished using an optical intensity of $I_{min} = 30$ $\hbox {mW}/\mu \hbox {m}^2$. This is within the range of diffraction-limited single-pass biosensors, such as evanescent nanofibre^4^.

The three examples above make it clear that the optical intensities required to observe different molecular dynamics can vary by many orders of magnitude from tens-of-milliwatts per square micron to megawatts per square micron. The lower range of these intensities can be reached in diffraction-limited single-pass biosensors such as those reported in Ref.^4^. Higher intensities would require the use of plasmonic or optical resonances^11,12,64,65^, which have already been successfully applied to observe enzyme-reactant reactions and conformational changes of DNA polymerase^25^ and, very recently and most relevant, conformational changes of single proteins^56^.

Plasmonic double nano-hole detectors have been operated at input powers that allow intensities of 5–50 $\hbox {W}/\upmu \hbox {m}^2$ at their hotspots^56,64^, while optoplasmonic whispering gallery mode sensors have been used to detect single BSA molecules with intensities of up to 5 $\hbox {W}/\upmu \hbox {m}^2$ at their hotspots^12^. Moreover, the signal-to-noise of these sub-diffraction limited sensors is also enhanced due to the high gradient of the illumination field. The optical scattering can then be modified after a conformational change not only by changes in molecular polarizability, but also by changes in the field intensity distribution across the molecule. Together, it appears feasible that shot-noise-limited plasmonic and optoplasmonic sensors could reach the required intensity levels to detect transitions between conformations in $\text {F}_1\text {F}_0$ ATPase, 26S proteasome and related molecules, consistent with the recent observations in Ref.^56^. On the other hand, our work predicts that thermally driven conformational fluctuations, such as we modelled for BSA, will remain unresolvable without the development of new substantially more sensitive technology.

### Impact of optical intensity on biological processes

The above calculations suggest that relatively high optical intensities may be required to resolve single molecule dynamics on timescales relevant to their conformational changes. However, high light intensities have been demonstrated to impact the structure and function of biological samples. Here we briefly overview some of these types of optical intrusion, the intensity levels at which they occur, and how they might impact measurements of conformational changes.

Intensities in the range of tens of Watts-per-micrometre-squared have been observed to cause protein unfolding and other changes in molecular structure and function. For instance, Pang et al. observe a two-step trapping intensity for BSA molecules in a double nano-hole plasmonic optical trap for intensities at a few Watts-per-micrometre-squared^11^, and conclude that the extra step is due to light-induced protein unfolding from the molecules normal state to a partially unfolded fast state. This is assuming a plasmonic trap similar to the one used by Ying et al. (a plasmonic intensity enhancement factor of 400, and a trap temperature that scales linearly with input power)^56^. Similarly, BSA irreversibly unfolds at temperatures above $60\,^{\circ }\hbox {C}$^66^. This corresponds to intensities of around 10 $\hbox {W}/\upmu \hbox {m}^2$ for a plasmonic trap sensor. The presence of light can also cause aggregation of molecules, and alter the function of molecular machines. For instance, aggregation has been observed to occur at light intensities of 1 $\hbox {W}/\upmu \hbox {m}^2$ for $\alpha$-chymotrypsin molecules attached to gold nanoparticles in solution^67^, while the function of ATPase is known to degrade at temperatures above $60\,^{\circ }$C^68^, corresponding to an intensity of about $10\,\hbox {W}/\upmu \hbox {m}^2$ for a plasmonic trap sensor.

Given these observations, it is clear that even if it was possible to reach the megawatts-per-square-micron intensities required to resolve thermally driven fluctuations of molecules such as BSA, such intensities would intrude prohibitively on the molecule. On the other hand our calculations suggest that steps between 26S proteasome conformations could be resolved with intensities that are well below known thresholds for optical intrusion (30 $\hbox {mW}/\upmu \hbox {m}^2$). The rotational steps of ATPase lie somewhere between these two extremes. The few-hundred Watt-per-micron-squared intensities we predict to be required to resolve these steps are around a factor of 10 above known thresholds for photodamage, but comparable to intensities that have been used to extract biophysically relevant information from biomolecules previously (e.g.^25,56^. Thus, it is possible that state-of-the-art unlabelled single molecule biosensors could resolve the stepping of ATPase, though at intensities that might affect the dynamics of the molecule.

It is interesting to ask whether the large polarization forces due to the high intensities involved in detecting single molecule dynamics could also themselves perturb the structure of the molecule. There are two contributions to the deformation of a molecule in a constant electric field^69^, the electrostatic compression due to field itself and the intrinsic deformation due to the induced-dipole created in the molecule. In the case of a single molecule, the second contribution is negligible as the dipole induced field is around $10^{15}$ to $10^{17}$ time smaller than the incident field. Approximating the protein molecule as a homogeneous sphere, we are able to estimate the level of deformation from electrostatic compression. In this case, the strain *S* applied to the molecule can be expressed as $S=M|\mathbf{E} |^2$ with $M=\varepsilon /Y$ the Maxwell electromechanical coefficient, $\varepsilon$ the molecule permittivity and *Y* the molecules Young modulus^69^. Taking $Y=1$ GPa, as a typical value for a single protein^70^, the mean intensity of a Gaussian beam required to deform a molecule by 10% (corresponding to a strain of $S=0.1$) is around $30\,\hbox {mW}/\upmu \hbox {m}^2$. This intensity is below the typical intensities needed to detect conformation changes. Therefore, molecules can be expected to undergo constant deformation during detection. We would emphasise, however, that such deformations do not preclude detection of conformational changes, as evidenced by the experimental results of Ref.^56^.

## Conclusion

We have introduced a new efficient method to calculate the dynamic polarizability of macromolecules from their atomic configuration. This method allows the polarizability to be calculated for molecules with size exceeding one hundred thousand atoms. The molecular conformations required as input can be taken from structure repositories such as the Protein Data Bank, or alternatively from molecular dynamics simulations. By providing a quantitative connection between variations in the polarizability of a protein and its underlying structural dynamics, our method provides a tool that could be used to better interpret single molecule experimental studies of important biological processes such as enzyme catalysis and protein folding.

## Supplementary Information

Supplementary Information.

## Data Availability

The code developed for this paper, using both sparse matrices and iterative matrix inversion, is available at Ref.^35^.
