# Supplementary material for: Modelling of the dynamic polarizability of macromolecules for single-molecule optical biosensing
Source: Sci Rep. 2022 Feb 7;12:1995. doi: 10.1038/s41598-022-05586-0 (PMC8821610; doi:10.1038/s41598-022-05586-0)
Supplement: Supplementary file 1 — Supplementary Information. [file 41598_2022_5586_MOESM1_ESM.pdf]

SUPPORTING INFORMATION

Modelling of the dynamic polarizability of macromolecules for single-molecule optical biosensing

Larnii S. Booth,[1] Eloise V. Browne,[1] Nicolas P. Mauranyapin,[1] Lars S. Madsen,[1] Shelley Barfoot,[2] Alan Mark,[2] and Warwick P. Bowen[1]

[1)] *ARC Centre for Engineered Quantum Systems (EQUS), School of Mathematics and Physics, The University of Queensland, Australia*

[2)] *School of Chemistry and Molecular Biosciences, The University of Queensland, Australia*

(Dated: 30 November 2021)

I. MOLECULAR DYNAMICS METHODS

The initial conformation of Bovine Serum Albium (BSA) was taken from PDB ID 3V03[1]. The ligand was removed and missing atoms modelled using PyMOL (www.pymol.org). The protein was placed in a periodic octahedral box with the minimum distance between the protein and the edge of the box being 1.2 nm. The system was solvated in explicit water. The GROMOS 54A7 united-atom force field[2–4] was used to describe the protein. TIP5P, a 5-point water model, was used to describe the solvent[5]. Lysine, arginine, and histidine residues were protonated mimicking neutral pH (7.0). No counterions were added. In order to relax the geometry of the protein, energy-minimisation was performed using harmonic constraints between bonds using the steepest decent approach.

The production simulations were run in triplicate using GROMACS 5.1.3[6]. Initial velocities were taken from a Maxwell-Boltzmann distribution at 300 K. Bond lengths within the protein were constrained using the LINCS algorithm[7]. The geometry of the water was constrained using SETTLE. To maintain a constant temperature, the protein and water were independently weakly coupled to an external temperature bath (300 K) with a relaxation time of 0.1 ps[8]. Isotropic pressure coupling was applied (reference pressure = 1.0 bar; relaxation time = 1.0 ps; compressibility = 4.5×10^{-5} bar^{-1})[8]. The pair-list was updated every 5 steps. The van der Waals cutoff was 1.4 nm[9]. Long-range electrostatic interactions were computed using Particle-mesh Ewald[10,11]. The step size was 2 fs. Configurations of the system were taken every 50 ps, and the systems were run for 54 ns each.

REFERENCES

[1] K. A. Majorek, P. J. Porebski, A. Dayal, M. D. Zimmerman, K. Jablonska, A. J. Stewart, M. Chruszcz, and W. Minor, "Structural and immunologic characterization of bovine, horse, and rabbit serum albumins," Molecular Immunology **52**, 174–182 (2012).

[2] N. Schmid, A. P. Eichenberger, A. Choutko, S. Riniker, M. Winger, A. E. Mark, and W. F. van Gunsteren, "Definition and testing of the gromos force-field versions 54a7 and 54b7," European Biophysics Journal **40**, 843–856 (2011).

[3] D. van der Spoel, E. Lindahl, B. Hess, G. Groenhof, A. E. Mark, and H. J. C. Berendsen, "Gromacs: Fast, flexible, and free," Journal of Computational Chemistry **26**, 1701–1718 (2005).

[4] D. Poger, W. F. van Gunsteren, and A. E. Mark, "A new force field for simulating phosphatidylcholine bilayers," Journal of Computational Chemistry **31**, 1117–1125 (2010).

[5] M. W. Mahoney and W. L. Jorgensen, "A five-site model for liquid water and the reproduction of the density anomaly by rigid, nonpolarizable potential functions," The Journal of Chemical Physics **112**, 8910–8922 (2000).

[6] M. J. Abraham, T. Murtola, R. Schulz, S. Páll, J. C. Smith, B. Hess, and E. Lindahl, "Gromacs: High performance molecular simulations through multi-level parallelism from laptops to supercomputers," SoftwareX **1–2**, 19–25 (2015).

[7] B. Hess, H. Bekker, H. J. C. Berendsen, and J. G. E. M. Fraaije, "Lincs: A linear constraint solver for molecular simulations," Journal of Computational Chemistry **18**, 1463–1472 (1997).

[8] H. J. C. Berendsen, J. P. M. Postma, W. F. van Gunsteren, A. DiNola, and J. R. Haak, "Molecular dynamics with coupling to an external bath," The Journal of Chemical Physics **81**, 3684–3690 (1984).

[9] S. Reißer, D. Poger, M. Stroet, and A. E. Mark, "Real cost of speed: The effect of a time-saving multiple-time-stepping algorithm on the accuracy of molecular dynamics simulations," Journal of Chemical Theory and Computation **13**, 2367–2372 (2017).

[10] T. Darden, D. York, and L. Pedersen, "Particle mesh ewald: An nlog(n) method for ewald sums in large systems," Journal of Chemical Physics **98**, 10089–10092 (1993).

[11] U. Essmann, L. Perera, M. L. Berkowitz, T. Darden, H. Lee, and L. G. Pedersen, "A smooth particle mesh ewald method," The Journal of Chemical Physics **103**, 8577–8593 (1995).
